# 采用高通量测序技术鉴别免疫球蛋白重链可变区体细胞高突变复杂克隆背景的研究

**DOI:** 10.3760/cma.j.cn121090-20241016-00394

**Published:** 2025-09

**Authors:** 梦鸽 高, 蓉 魏, 扬 刘, 晓军 黄, 申淼 杨, 晓甦 赵

**Affiliations:** 北京大学人民医院，北京大学血液病研究所，国家血液系统疾病临床医学研究中心，造血干细胞移植治疗血液病北京市重点实验室，北京 100044 Peking University People's Hospital, Peking University Institute of Hematology, National Clinical Research Center for Hematologic Disease, Beijing Key Laboratory of Hematopoietic Stem Cell Transplantation, 100044 Beijing, China

**Keywords:** 免疫球蛋白重链可变区基因, 体细胞高突变, Sanger测序, 高通量测序, Immunoglobulin heavy chain variable gene, Somatic hypermutation, Sanger sequencing, Next generation sequencing

## Abstract

**目的:**

比较高通量测序（NGS）与Sanger测序在评估免疫球蛋白重链可变区（IGHV）基因体细胞高突变（SHM）状态中的应用差异，并着重探讨导致两种技术结果不一致的关键因素（尤其在复杂克隆背景下）。

**方法:**

回顾性分析2016–2021年于北京大学人民医院收集的Sanger测序结果显示为非单克隆（43例）和单克隆（10例）的淋系肿瘤样本，并采用NGS对其进行IGHV SHM检测。将两种方法的结果进行系统性比对，并从克隆丰度量化、引物设计差异及解读标准等角度，对结果不一致的病例进行分析。

**结果:**

53例同时进行Sanger测序及NGS检测的患者中，男36例，女17例，中位年龄64（33～88）岁。慢性淋巴细胞白血病35例（66.0％）、弥漫大B细胞淋巴瘤9例（17.0％）、滤泡性淋巴瘤3例（5.7％）、套细胞淋巴瘤3例（5.7％）、其他3例（5.7％）。在43例Sanger测序结果为非单克隆背景的样本中，NGS检测结果显示23例为双克隆或多克隆，17例为单克隆，3例未检出克隆性。Sanger与NGS结果的主要差异体现在克隆性评估、IGHV基因重排类型以及突变率方面。在10例Sanger测序结果为单克隆的病例中，NGS检测出2例双克隆，另有4例的IGHV重排类型与Sanger结果差异明显。尽管两种方法在SHM比例检测上存在细微差异，但对突变状态的整体判断无实质影响。

**结论:**

与Sanger测序相比，NGS在IGHV SHM的复杂克隆背景中更具优势，尤其在识别亚克隆成分和精确量化克隆比例方面更具敏感性和准确性，可为慢性淋巴细胞白血病等淋系肿瘤的精准诊断及预后评估提供更为精准的分子依据。

## Abstract (en)

**Objective:**

To compare the performance of next-generation sequencing（NGS）and Sanger sequencing in investigating somatic hypermutation（SHM）status of immunoglobulin heavy chain variable region（IGHV）genes. It specifically focuses on identifying key factors contributing to discrepancies between the two methods, particularly under complex clonal backgrounds, to inform optimized strategies for clinical application.

**Methods:**

This retrospective analysis included 53 samples, comprising 43 identified as non-monoclonal and 10 as monoclonal using Sanger sequencing. All samples were further analyzed using NGS to assess IGHV SHM. The two methods were used for systematic comparison. For discordant cases, in-depth attribution analysis was conducted, considering factors, including clonal abundance quantification, differences in primer design, and interpretation criteria.

**Results:**

Among the 53 patients who underwent both Sanger and NGS testing, 36 were male and 17 were female, with a median age of 64 years（range: 33–88）. Diagnoses included chronic lymphocytic leukemia（CLL）in 35（66.0％）, diffuse large B-cell lymphoma in 9（17.0％）, follicular lymphoma in 3（5.7％）, mantle cell lymphoma in 3（5.7％）, and other types in 3（5.7％）cases. In the 43 cases with non-monoclonal profiles using Sanger sequencing, NGS revealed 23 cases as biclonal or polyclonal, 17 as monoclonal, and 3 with no detectable clonality. The primary discrepancies between the two methods involved variations in clonality assessment, IGHV gene rearrangement types, and mutation rates. Among the 10 cases identified as monoclonal using Sanger sequencing, NGS detected biclonality and markedly different IGHV rearrangement types in 2 and 4 cases, respectively. Minor differences were observed in SHM percentage between the two methods; however, these did not substantially affect the overall determination of mutational status.

**Conclusion:**

Compared with Sanger sequencing, NGS exhibits superior performance in assessing IGHV SHM status under complex clonal conditions. It provides greater sensitivity and accuracy in detecting subclonal components and quantifying clonal proportions, thereby providing a more precise molecular basis for diagnosing and prognostically assessing lymphoid malignancies, including CLL.

慢性B淋巴细胞增生性疾病是一类成熟淋巴细胞特征的克隆性增生性疾病[1]–[2]。在正常B淋巴细胞的发育与分化过程中，免疫球蛋白重链（IGH）的可变（V）、多样性（D）和连接（J）区域基因在生殖系状态下发生重排。在淋巴组织的生发中心，当B细胞暴露于抗原时，IGH V-D-J基因重排可引发体细胞高突变（SHM），主要体现在可变互补决定区（CDR）核苷酸的变化[3]–[4]。由于重排基因片段的随机性和多样性，每个克隆的B细胞均具有独特的基因重排模式，成为B淋巴细胞克隆的基因标志物[5]–[6]。目前研究表明，无突变型慢性淋巴细胞白血病（CLL）通常预后较差，且对化疗和免疫治疗的敏感性较低，而突变型则预后较好[7]–[13]。这可能与SHM产生的免疫球蛋白与抗原结合能力强有关。然而，某些特定亚型（如IGHV3-21重排）无论SHM状态如何，预后均较差[6],[14]–[15]。因此，准确识别IGHV克隆的重排类型及其突变状态对CLL风险分层至关重要。

传统的IGHV SHM检测主要采用Sanger测序，通过将检测到的IGHV基因序列与生殖系序列进行比较，计算SHM频率。尽管Sanger测序被视为金标准[5],[7]–[9]，但Sanger测序难以准确检测多克隆重排，并且无法量化单一克隆的相对丰度，低估了SHM发生率且限制了对克隆性谱系的深入解析[5],[8]。此外，该方法操作繁琐，适用的病例数量也有限。

近年来，随着高通量测序（NGS）技术的进步，克隆序列的识别与克隆丰度定量的分析显著改善。该技术通过扩增子方法构建文库，识别多克隆重排序列，实现精确鉴定与量化[5],[8]。NGS能够克服Sanger测序在IGHV SHM分析中的局限性，无差别地检测所有IGHV克隆重排，明确特定克隆的序列及其相对比例，从而改善CLL的分层与管理[5],[11],[16]–[17]。此外，NGS技术高效处理大量病例，有助于在实验室中推广应用。

然而，目前关于Sanger测序与NGS在淋系肿瘤患者中IGHV SHM检测临床应用性能的对比研究仍较有限，尤其在存在双克隆或多克隆的复杂背景下。因此，本研究旨在系统评估两种方法在IGHV突变状态判定、优势克隆识别及克隆丰度定量分析中的表现，并比较它们在复杂克隆背景患者中的技术优势。

## 病例与方法

1. 病例：本研究为单中心横断面研究。我们回顾性分析了2016年至2021年间在我院通过Sanger测序评估IGHV状态的淋系肿瘤患者外周血或骨髓样本。在具有Sanger测序的非单克隆病例中，仅有43例样本在回顾时仍保留有足够的标本，得以进一步进行NGS检测。与此同时，我们选取了10例Sanger测序单克隆病例样本作为对照，最终共纳入53例进行NGS分析。本研究设计遵循《赫尔辛基宣言》的原则，并已获得医院伦理委员会的批准（2016PHA038）。

2. DNA提取与鉴定：使用EDTA抗凝的患者骨髓或外周血样本，通过红细胞裂解液离心获得有核细胞。使用DNA提取试剂盒提取DNA，并利用分光光度计检测DNA浓度和纯度。使用Qubit系统（Thermo Fisher公司产品，美国）进行DNA浓度测定。

3. Sanger测序：根据IdentiClone™重排试剂盒（Invivoscribe公司产品，美国）的说明，对患者标本的IGH基因重排进行PCR扩增。PCR条件：95 °C预变性7 min，接着35个循环：95 °C 45 s，60 °C 45 s，72 °C 90 s，最后72 °C延伸10 min。取1 µl的PCR扩增产物，加入10 µl甲酰胺和0.1 µl GeneScan-500 LIZ（Applied Biosystems公司产品，美国），95 °C热变性2 min后，储存于4 °C。使用3130基因分析仪（Applied Biosystems公司产品，美国）进行分析。

4. NGS检测方法：通过扩增子法构建文库以检测IGH基因重排克隆。使用LymphoTrack IGH Assay-PGM/S5® Kit试剂盒（Invivoscribe公司产品，美国）进行PCR扩增。PCR条件：95 °C预变性7 min，接着29个循环：95 °C 45 s，60 °C 45 s，72 °C 90 s，最后72 °C延伸10 min。文库使用AMPure XP beads（Beckman公司产品，美国）纯化，并使用KAPA文库定量试剂盒（Thermo Fisher公司产品，美国）进行定量。根据定量结果和所需的总读数，收集并稀释文库。模板通过Ion Chef系统制备，并在Ion S5平台（Thermo Fisher，美国）上测序，生成FASTQ文件。

5. 数据分析与定义：使用Lympho Track软件（Invivoscribe公司产品，美国）进行数据分析。由于没有公认的标准定义亚克隆的阈值，本研究参考Stamatopoulos等[2]的研究方法，仅第一序列总检测片段条数（total reads）≥2.5％被定义为单克隆重排；前两序列总检测片段条数均≥2.5％，且至少为第三序列的2倍，或前三序列比例相近且均≥2.5％则分别被定义为双克隆和多克隆重排。与胚系IGHV序列相比，相似性<98％的IGHV序列被定义为IGHV有突变，相似性≥98％被定义为IGHV无突变[11]。

## 结果

一、患者特征

53例同时有Sanger测序及NGS结果的患者中，男36例，女17例，中位年龄64（33～88）岁。疾病类型：CLL 35例（66.0％）、弥漫大B细胞淋巴瘤9例（17.0％）、滤泡性淋巴瘤3例（5.7％）、套细胞淋巴瘤3例（5.7％）、其他3例（5.7％）。53例患者进行NGS检测的样本中，41例（77.4％）为外周血，12例（22.6％）为骨髓。

二、Sanger测序与NGS检测结果比对

根据Sanger测序结果，43例为非单克隆，10例为单克隆。NGS分析结果显示，25例（47.2％）为双克隆/多克隆，25例（47.2％）为单克隆，另有3例（5.6％）未检测到克隆。

在43例Sanger测序为非单克隆样本中，NGS检测到3例无克隆和17例单克隆，该20例IGHV SHM不一致病例NGS结果见表1。在例1～4中，NGS检测的前三个序列的总读数比例较为接近，而在例5～20中序列总读数比例差异较大。

**表1:** Sanger测序非单克隆病例中Sanger测序和NGS结果不一致的对比分析

例号	Sanger测序克隆性	NGS				Sanger测序与NGS结果差异类型
		克隆性	SHM（％）	IGHV基因	前三个克隆序列读数（％）	
1	非单克隆	无克隆	NA	NA	2.31/0.57/0.39	克隆性差异
2	非单克隆	无克隆	NA	NA	1.40/1.07/1.06	克隆性差异
3	非单克隆	无克隆	NA	NA	0.68/0.66/0.66	克隆性差异
4	非单克隆	单克隆	8.41	4-59*07	5.26/3.73/2.13	克隆性差异
5	非单克隆	单克隆	5.29	3-74*01	10.72/2.71/2.41	克隆性差异
6	非单克隆	单克隆	7.49	3-23*04	52.42/1.29/0.84	克隆性差异
7	非单克隆	单克隆	2.41	4-61*01	65.75/0.63/0.31	克隆性差异
8	非单克隆	单克隆	9.83	4-39*01	16.83/1.05/0.94	克隆性差异
9	非单克隆	单克隆	8.37	3-74*03	21.57/3.57/2.92	克隆性差异
10	非单克隆	单克隆	8.11	3-53*02	66.79/0.16/0.12	克隆性差异
11	非单克隆	单克隆	7.02	4-34*02	61.12/2.51/2.14	克隆性差异
12	非单克隆	单克隆	4.84	3-7*03	55.73/2.01/1.73	克隆性差异
13	非单克隆	单克隆	0	3-11*01	65.51/1.28/0.21	克隆性差异
14	非单克隆	单克隆	0	3-30*04	76.45/0.29/0.19	克隆性差异
15	非单克隆	单克隆	3.51	4-4*07	12.03/1.16/0.87	克隆性差异
16	非单克隆	单克隆	15.93	7-4-1*02	80.65/0.73/0.14	克隆性差异
17	非单克隆	单克隆	9.25	3-7*01	24.62/1.08/0.49	克隆性差异
18	非单克隆	单克隆	6.84	4-39*02	82.64/0.28/0.20	克隆性差异
19	非单克隆	单克隆	7.02	4-34*02	84.47/0.03/NA	克隆性差异
20	非单克隆	单克隆	6.85	4-34*06	68.67/0.89/0.48	克隆性差异

**注** NGS：高通量测序；SHM：体细胞高突变；IGHV：免疫球蛋白重链可变区；NA：不适用

为进一步比较两种方法在单克隆样本中的表现，我们分析了10例Sanger测序为单克隆的病例。表2显示，两者不一致主要体现在以下三方面：①克隆性差异，Sanger测序检测到单克隆，而NGS检测到双克隆（例21～22）；②IGHV重排基因不同（例23～26）；③两种方法检测的SHM比例不同，但这不会影响突变状态（突变或非突变）的评估（例27～30）。总体而言，在Sanger测序单克隆病例中，NGS的结果较Sanger测序存在细微差异，但这些差异不会显著影响SHM的评估。

**表2:** Sanger测序单克隆病例中Sanger测序与NGS结果的对比分析

例号	Sanger测序			NGS				Sanger测序与NGS结果差异类型
	克隆性	SHM（％）	IGHV基因	克隆性	SHM（％）	IGHV基因	前三个克隆序列读数（％）	
21	单克隆	0.7	5-51*01	双克隆	0.89/0	5-51*03/2-70*01	43.07/29.63/0.26	克隆性差异
22	单克隆	9.9	3-21*02	双克隆	7.93/11.45	3-21*02/3-21*02	38.60/32.12/0.35	克隆性差异
23	单克隆	1.7	4-34*01	单克隆	0	4-34*02	59.50/2.19/1.40	IGHV重排基因差异
24	单克隆	0	3-21*01	单克隆	0	3-21*02	53.98/1.40/1.10	IGHV重排基因差异
25	单克隆	1.8	4-39*05	单克隆	3.95	4-34*02	55.05/0.69/0.44	IGHV重排基因差异
26	单克隆	3.2	3-23*01	单克隆	0	3-7*01	71.19/1.22/0.28	IGHV重排基因差异
27	单克隆	10.3	3-7*01	单克隆	10.57	3-7*01	71.81/0.60/0.33	突变频率差异
28	单克隆	0.4	4-39*01	单克隆	0	4-39*01	65.65/0.21/0.13	突变频率差异
29	单克隆	6.7	4-39*01	单克隆	8.55	4-39*01	61.15/0.64/0.53	突变频率差异
30	单克隆	0	3-11*05	单克隆	0	3-11*05	70.67/1.06/0.54	突变频率差异

**注** NGS：高通量测序；SHM：体细胞高突变；IGHV：免疫球蛋白重链可变区

## 讨论

本研究通过NGS检测分析了Sanger测序显示非单克隆及单克隆的病例，揭示了两种技术在结果解读和技术性能上的差异。Sanger测序由于其固有的技术局限性，在某些临床情境下无法提供准确的结果，从而阻碍临床决策。相比之下，NGS在克隆谱系分析和微小克隆检测方面优于Sanger测序，有助于更深入地理解CLL的发病机制[18]–[23]。

Sanger测序与NGS结果存在差异可能有以下原因：

首先，差异可能与解释标准有关。Sanger测序因无法准确量化单一克隆的相对丰度，在某些情况下会显示双克隆或多克隆背景，而NGS检测可能报告为无克隆性或单克隆（例1～4）。例如，当NGS中主要克隆序列的读取量低于2.5％，且前三个克隆序列比例接近时，Sanger测序可能显示为多克隆背景，而NGS则可能为无克隆性。此外，还有个别病例Sanger测序显示单克隆，但NGS检测为双克隆（例21～22），这可能与前三个克隆序列的总读取比例差异较大，且前两个序列读取比例远高于第三个序列有关。

其次，两种方法在引物设计上的差异也是造成结果不一致的重要原因。Sanger测序采用位于leader区的引物，能够扩增出完整的IGHV序列，更适合用于与种系基因的相似性比对；而NGS通常使用框架区1（FR1）引物[5],[8],[17]，更侧重于提高检测效率。由于FR1引物无法覆盖上游约60个核苷酸序列，可能导致突变率略高，但这一差异一般不会影响临床判断[2],[5],[8]–[9],[11]。此外，引物差异还可能影响IGHV重排等位基因的检测结果。尤其在NGS中，每个IGHV家族通常仅使用1个通用的FR1引物，若引物结合区存在个体间的序列多态性，可能导致部分等位基因未被有效扩增，进而影响检测结果的全面性和准确性。

综上所述，本研究展示了Sanger测序的技术局限性和NGS在IGHV SHM复杂克隆背景评估中的优势。然而，NGS与Sanger测序在部分病例中仍存在结果不一致的情况，提示其临床应用尚需进一步验证。未来应结合更多样本的系统研究和长期随访，明确NGS在不同临床场景下的应用价值和适用边界。此外，对于少见或复杂的IGHV重排模式，仍需制定更统一的解读标准与实践指南，以提高NGS结果在不同实验室和临床中心间的可比性和可推广性，推动其在临床中的规范化应用。
